# Supplementary material for: Vitamin D Modified DSS-Induced Colitis in Mice via STING Signaling Pathway
Source: Biology (Basel). 2025 Jun 18;14(6):715. doi: 10.3390/biology14060715 (PMC12190092; doi:10.3390/biology14060715)
Supplement: Supplementary file 1 [file biology-14-00715-s001.zip › Supplementary Materials S1: 16s-report of gut microbiota/Groups/diff_analysis/UnivarTestMETACYC/figure/barplot/top20log.pdf]

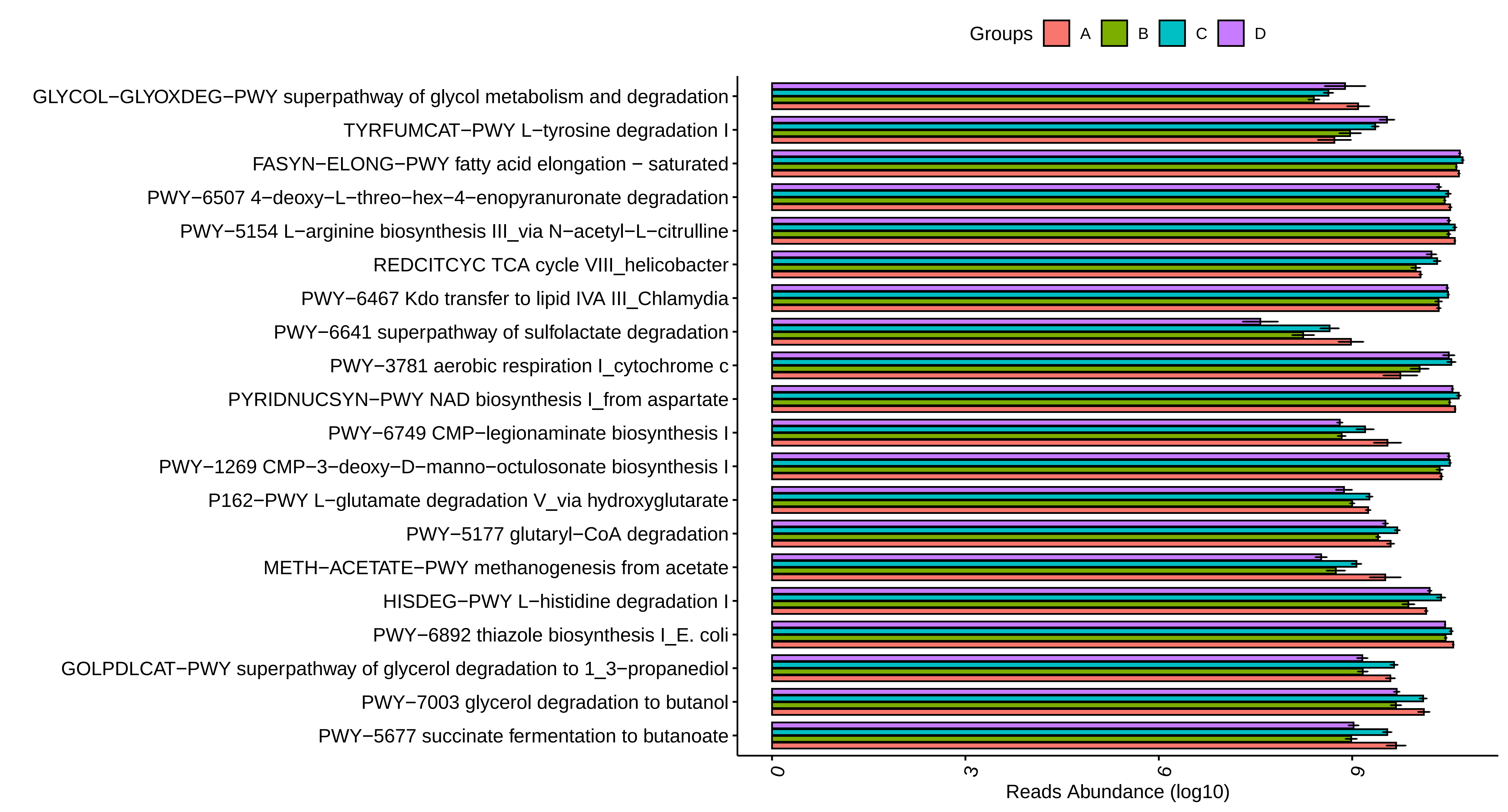

Groups
A
B
C
D
GLYCOL−GLYOXDEG−PWY superpathway of glycol metabolism and degradation
TYRFUMCAT−PWY L−tyrosine degradation I
FASYN−ELONG−PWY fatty acid elongation − saturated
PWY−6507 4−deoxy−L−threo−hex−4−enopyranuronate degradation
PWY−5154 L−arginine biosynthesis III_via N−acetyl−L−citrulline
REDCITCYC TCA cycle VIII_helicobacter
PWY−6467 Kdo transfer to lipid IVA III_Chlamydia
PWY−6641 superpathway of sulfolactate degradation
PWY−3781 aerobic respiration I_cytochrome c
PYRIDNUCSYN−PWY NAD biosynthesis I_from aspartate
PWY−6749 CMP−legionaminate biosynthesis I
PWY−1269 CMP−3−deoxy−D−manno−octulosonate biosynthesis I
P162−PWY L−glutamate degradation V_via hydroxyglutarate
PWY−5177 glutaryl−CoA degradation
METH−ACETATE−PWY methanogenesis from acetate
HISDEG−PWY L−histidine degradation I
PWY−6892 thiazole biosynthesis I_E. coli
GOLPDLCAT−PWY superpathway of glycerol degradation to 1_3−propanediol
PWY−7003 glycerol degradation to butanol
PWY−5677 succinate fermentation to butanoate
0
3
6
9
Reads Abundance (log10)
